# Natal Host Plants Can Alter Herbivore Competition

**DOI:** 10.1371/journal.pone.0169142

**Published:** 2016-12-28

**Authors:** Huipeng Pan, Evan L. Preisser, Qi Su, Xiaoguo Jiao, Wen Xie, Shaoli Wang, Qingjun Wu, Youjun Zhang

**Affiliations:** 1 Department of Entomology, South China Agricultural University, Guangzhou, China, Key Laboratory of Bio-Pesticide Innovation and Application, Engineering Technology Research Center of Agricultural Pest Biocontrol of Guangdong Province, Guangzhou, China; 2 Department of Plant Protection, Institute of Vegetables and Flowers, Chinese Academy of Agricultural Sciences, Beijing, China; 3 Biological Sciences Department, University of Rhode Island, Kingston, Rhode Island, United States of America; Helmholtz Zentrum Munchen Deutsches Forschungszentrum fur Umwelt und Gesundheit, GERMANY

## Abstract

Interspecific competition between herbivores is widely recognized as an important determinant of community structure. Although researchers have identified a number of factors capable of altering competitive interactions, few studies have addressed the influence of neighboring plant species. If adaptation to/ epigenetic effects of an herbivore’s natal host plant alter its performance on other host plants, then interspecific herbivore interactions may play out differently in heterogeneous and homogenous plant communities. We tested wether the natal host plant of a whitefly population affected interactions between the Middle-east Asia Minor 1 (MEAM1) and Mediterranean (MED) cryptic species of the whitefly *Bemisia tabaci* by rearing the offspring of a cabbage-derived MEAM1 population and a poinsettia-derived MED population together on three different host plants: cotton, poinsettia, and cabbage. We found that MED dominated on poinsettia and that MEAM1 dominated on cabbage, results consistent with previous research. MED also dominated when reared with MEAM1 on cotton, however, a result at odds with multiple otherwise-similar studies that reared both species on the same natal plant. Our work provides evidence that natal plants affect competitive interactions on another plant species, and highlights the potential importance of neighboring plant species on herbivore community composition in agricultral systems.

## Introduction

What determines the outcome of herbivore competition? As interspecific herbivore competition became recognized as both widespread and important [1], ecologists identified a number of potentially influential factors. Within a trophic level, the ability to survive on lower-quality resources, grow more quickly on a given resource, or decrease resource quantity/quality for a later-arriving competitor are important; across trophic levels, the role of predator-mediated apparent competition or induced plant defenses can also be critical [2]. Such plant-mediated interactions yield competition between herbivores feeding on different plant structures: there is now abundant evidence, for instance, that foliar- and root-feeding species can affect each other's growth and survival [3, 4].

While controlled experiments are necessary to identify the mechanisms driving interspecific herbivore competition, such approaches necessarily involve manipulating a few causative factors while holding others constant. Because even polyphagous herbivores exhibit host plant preferences, for example, experiments seeking to assess interspecific competition on a given host plant generally rear both herbivore species on that plant before allowing them to compete [5, 6]. While such a protocol facilitates a 'clean' comparison of herbivores' competitive interactions, it excludes the possibility that nearby plant species influence the outcome [7]. Such 'neighborhood' effects have been found to affect herbivores in a number of ways. Associational susceptibility or resistance, for example, occur when plants growing near another plant species experience more or less herbivory, respectively [8]. This can occur by both altered apparency as well as defenses induced by another species' volatile cues [7].

Despite our rapidly-growing appreciation of neighboring species' importance to focal plant fitness, there has been relatively little exploration of how such effects might affect herbivores. To give one example, the offspring of a polyphagous herbivore feeding on one host might settle on another nearby plant species and compete with its resident herbivores; would the herbivores' origin influence their growth, survival, and interspecific interactions? There is evidence that the offspring of herbivores reared on different varieties of a particular host plant can do better on that variety, either via adaptation to that host or a phenomena referred to as ‘transgenerational acclimatization’. The offspring of *Coenonympha pamphilus* butterflies reared on low-nitrogen *Festuca rubra*, for instance, did better on these hosts than larvae whose parents were reared on high-nitrogen *F*. *rubra* [9]. More generally, maternal effects are well known to affect offspring fitness via epigenetic or other mechanisms [10], and their impact can extend across two or even three generations [11–13]. Although the adaptive advantages accruing to parents capable of 'optimizing' their lineages for survival on a particular host plant are clear, either adaption or transgenerational acclimatization may also improve performance on other host species.

We report the results of work demonstrating that an herbivore’s host plant can alter the outcome of interspecific competition. Specifically, we find that the result of interspecific competition between herbivores can be reversed when two cryptic species (MEAM1 and MED) of the whitefly *Bemisia tabaci* are reared on natal host plants different than the plant species on which they compete. Because many natural systems contain a mixture of plant species, this finding may have widespread implications.

## Materials and Methods

### Natural history of the system

The sweetpotato whitefly *Bemisia tabaci* (Gennadius) is a globally-distributed polyphagous herbivore that includes a number of genetically divergent but morphologically indistinguishable species [14]. The various *B*. *tabaci* species differ in a number of important aspects such as their host range, feeding behavior, vector competence, insecticide resistance, and endosymbiont community structure [15–20]. Two of these species, MEAM1 (formerly biotype ‘B’) and MED (formerly biotype ‘Q’), are major agricultural pests of agricultural ecosystems [21] found in over 60 countries worldwide [14].

The highly-invasive nature of both MEAM1 and MED, and their overlapping distributions, has led to numerous investigations of their competitive interactions [5, 6, 22, 23]. Interest in this topic has been heightened by the fact that lab experiments yield results different from those seen in the field: MEAM1 generally excludes MED in laboratory settings but has been excluded by MED in China and other Asian countries [24, 25]. Factors such as differential insecticidal resistance [5, 22, 23] and varying host plant preferences [15, 26] have been identified as possible non-exclusive explanations for this disparity.

### Whitefly populations and ancestral host plants

MEAM1 was originally collected in 2004 from cabbage, *Brassica oleracea* cv. Jingfeng1, growing in the Haidian District of Beijing, China. The MED population was originally collected in 2009 from poinsettia, *Euphorbia pulcherrima* Willd. ex Klotz., growing in the same region. In both cases, the collection was from plants grown on land belonging to the Institute of Vegetables and Flowers, Chinese Academy of Agricultural Sciences; as researchers at this institution, we were explicitly given permission to collect MEAM1 from these sites. None of the three species (MEAM1, *E*. *pulcherrima*, and *B*. *oleracea*) are endangered or protected species in China. Populations of each species were reared in separate screen cages under natural lighting and ambient temperature (26±2°C) in a glasshouse. To ensure that each population consisted of a single species, we sequenced the mitochondrial cytochrome oxidase 1 (*mtCO1*) gene marker [24] of 15 adults per generation per population.

MEAM1 and MED populations were maintained on potted cabbage and poinsettia, respectively. Plants were cultivated singly in a 1.5L pot filled with potting mix (peat moss, vermiculite, organic fertilizer, and perlite in a 10:10:10:1 ratio by volume). Prior to their exposure to whiteflies, all plants were held in whitefly-proof screen cages in a greenhouse under natural lighting and controlled temperature (26±2°C). Cabbage (*B*. *oleracea*, cv. Jingfeng 1) and cotton (*G*. *hirsutum* cv. DP99B) plants were used in the experiment when they had 5–7 fully-expanded true leaves; poinsettias were used when they were 25-30cm high.

### Experimental design

To test whether the initial host plant affected the results of MEAM1-MED competition on subsequent host plants, we inoculated cabbage, cotton, and poinsettia with MEAM1 reared on cabbage and MED reared on poinsettia. Each experimental replicate consisted of a single whitefly-proof, ventilated cage (0.6m x 0.4m x 0.8m) containing two individually-potted host plants. Each replicate was inoculated with 20 pairs of MEAM1 and 20 pairs of MED. The experiment was replicated four times using cabbage, three times using cotton, and five times using poinsettia. Each cage was then placed in a larger screen cage (to minimize the risk of cross-contamination) and held in a glasshouse under natural lighting and ambient temperature (26±2°C). Both the inner and outer cages of each replicate were kept sealed except when plants were watered or whitefly populations sampled (detailed below).

Every 25–27 days (~1 generation), 100 haphazardly-selected whiteflies were collected from each cage for species determination. Immediately after the 100 whiteflies were collected, we removed one of the two whitefly-infested plants (and all the whiteflies on it) in the cage and replaced it with a similarly-sized uninfested plant of the same species. This was done to prevent overcrowding. Sampling ended when only a single whitefly species was in a given cage. The genomic DNA was extracted from each whitefly according to White et al. [27], and stored at -20°C until analysis. The identity (MEAM1 or MED) of each individual was determined by the CAPS of *mtCOI* with the restriction endonucleases *Vsp*I [24]. We used this information to determine the percentage of MED individuals for each cage*sample*plant species combination.

### Statistical analysis

The unit of replication for all analyses was the percentage of MED per cage per time per plant species. We followed recommended procedures for percentage data and analyzed logit-transformed (value +0.01) data. Because cages were sampled repeatedly over time, an rm-ANOVA design was used to analyze whether the percentage of MED changed over time in each of the three treatments (= plant species, a fixed factor in the analysis). JMP v.9 was used for all analyses.

## Results

The mean percentage of MED differed in each of the three treatments (F_2,9_ = 9833, p < 0.001) and over time (F_8,2_ = 24720, p = 0.001). There was also a significant treatment*time interaction (F_16,4_ = 1201, p < 0.001), indicating that MED percentages in the three treatments changed differently over time.

**Fig 1:**
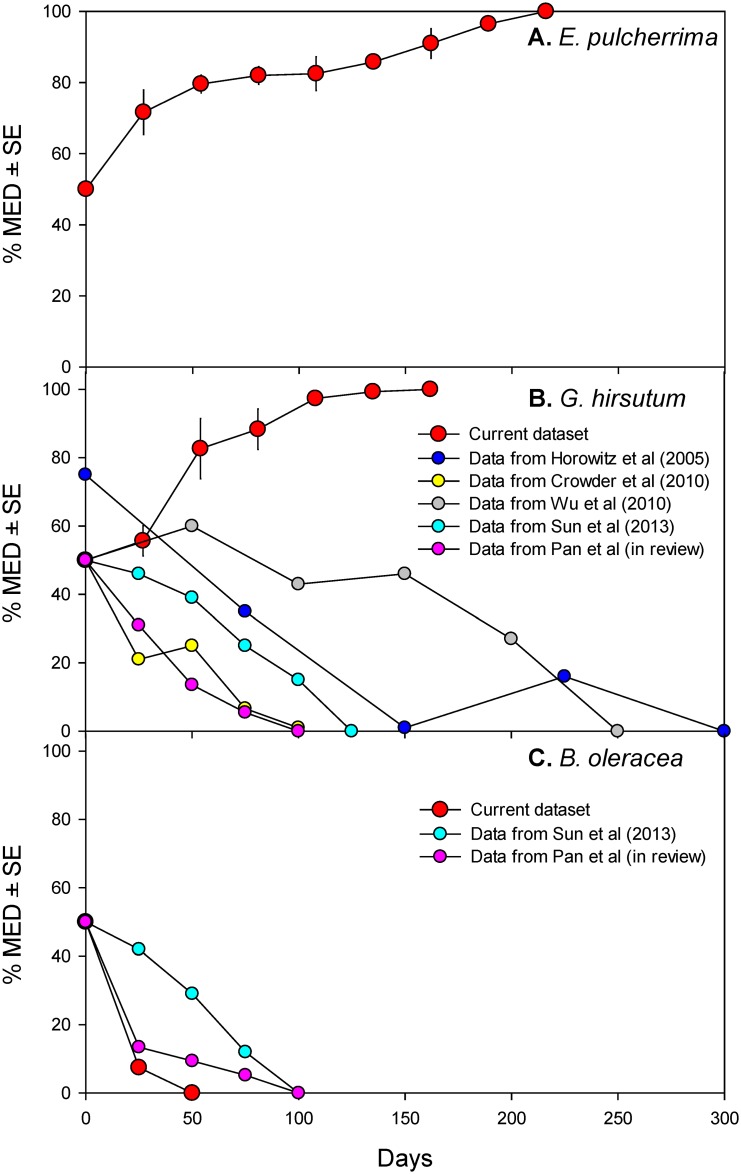
Percentage of MED on *E*. *pulcherrima*, *G*. *hirsutum*, and *B*. *oleracea*. **(A, top panel):** Percentage of MED on *E*. *pulcherrima*. *E*. *pulcherrima*-derived MED is competing with *B*. *oleracea* cv. Jingfeng 1-derived MEAM1; see text for treatment details. Values are mean (±SE) of the percentage of MED per replicate (N = 5). **(B, middle panel):** Percentage of MED on *G*. *hirsutum* cv. DP99B. Large red circles: data from this study on *E*. *pulcherrima*-derived MED competing with *B*. *oleracea* cv. Jingfeng 1-derived MEAM1; values are mean (±SE) of the percentage of MED per replicate (N = 3). Small circles: data from five studies in which MED and MEAM1 were reared on the same host plant and allowed to compete on *G*. *hirsutum*. In Horowitz et al [28], figure 5 in Crowder et al [5]; both MEAM1 and MED were reared and experimented on cv. Atala.; in Crowder et al [5], on cv. DP5415; in Wu et al [6], on cv. Simian-8; in Sun et al [23], on cv. Zhe-Mian 1793; in Pan et al [22], both MEAM1 and MED were reared on *L*. *esculentum* cv. Zhongza 9 and experimented on cv. DP99B. **(C, bottom panel):** Percentage of MED on *B*. *oleracea* cv. Jingfeng 1. Large red circles: data from this study on *E*. *pulcherrima*-derived MED competing with *B*. *oleracea*-derived MEAM1; values are mean (±SE) of the percentage of MED per replicate (N = 4). Small circles: Data from two additional studies in which MEAM1 and MED were reared on the same host plant and allowed to compete on *B*. *oleracea* cv. Jingfeng 1. In Sun et al [23], both were reared on *G*. *hirsutum* cv. Zhe-Mian 1793; in Pan et al [22], both were reared on *L*. *esculentum* cv. Zhongza 9.

When cabbage-derived MEAM1 and poinsettia-derived MED were reared together on poinsettia, MED excluded MEAM1 by the ninth sampling period in all of the experimental replicates (fig 1A). When cabbage-derived MEAM1 and poinsettia-derived MED were reared together on cotton, MED increased in abundance and excluded MEAM1 by the seventh sampling period in all of the experimental replicates (fig 1B). When cabbage-derived MEAM1 and poinsettia-derived MED were reared together on cabbage, MEAM1 excluded MED by the third sampling period (fig 1C).

## Discussion

We found that the offspring of poinsettia*-*derived MED competitively excluded the offspring of cabbage-derived MEAM1 when reared together on poinsettia (fig 1A) and on cotton (fig 1B). While ours is the first study to assess MEAM1-MED competition on poinsettia, the results from cotton run counter to the findings of multiple studies [5, 6, 22, 23, 28] that evaluated MEAM1-MED competition on cotton and found MEAM1 excluded MED. These studies are virtually identical to ours except in the choice of natal host plant: four reared both species on cotton beforehand [5, 6, 23, 28], and the fifth reared them on tomato [22]. The disparity between our results and theirs implicates our pre-experiment choice of natal host plant(s) as the factor responsible for altering the outcome of herbivore competition. The fact that competitive exclusion of MEAM1 by MED occurred over an ~150-day period, even though the generation time of both MEAM1 and MED on *G*. *hirsutum* is 20–25 days [29, 30], suggests that this result is unlikely to be explained by epigenetic changes linked to the whiteflies’ original host plant. For epigenetic changes to have produced our result, they would have had to persist for at least six generations. Although this possibility seems improbable, it is worth noting that the most rapid increase in MED frequency occurred within the first two generations of the experiment (fig 1B). This would be consistent with transgenerational effects that primarily affect the first and second generations; although these effects may dissipate afterwards, MED may by then possess such a large numerical advantage that it is able to displace the ‘competitively dominant’ MEAM1 [31].

While the natal host plant altered the outcome of MEAM1-MED competition on cotton, it did not have a similar effect on other host plants. When the offspring of poinsettia-derived MED and cabbage-derived MEAM1 were reared together on cabbage, MEAM1 quickly excluded MED (Fig 1C). This result agrees with other work that reared both species pre-experiment on cabbage [23] or tomato [22]. Our findings thus demonstrate how herbivore competition can be affected by each species’ natal host plant(s), the plant on which the species compete, and the interaction between these factors.

Our finding that MED excluded MEAM1 on poinsettia is consistent with previous research showing it is a much better host plant for MED than for MEAM1. Scientists investigating the poinsettia-driven ‘Christmas invasion’ of *B*. *tabaci* found that this plant often introduces MED into MEAM1-colonized areas [32], while populations of MEAM1 do better on vegetables than on poinsettia or other ornamental plants [33]. In a comparative study, Liu et al. [15] found that MED feeding on poinsettia had longer probe durations and ingested more phloem than MEAM1. When MEAM1 and MED were reared on *Cucumis sativa* and allowed to choose between host plants, MED preferred to settle and oviposit on poinsettia and cotton over cabbage, while MEAM1 preferred cabbage over poinsettia and cotton [34]. A subsequent no-choice experiment found that MED survival and fecundity was greater on poinsettia and cotton than cabbage, but that the opposite was true for MEAM1 [34].

Studies documenting the competitive exclusion of MED by MEAM1 on cotton have identified two factors as being primarily responsible for this outcome. First, MEAM1 appears to grow better on cotton than MED. A study comparing the two species’ performance found that while their fecundity and survival was similar, the developmental period of MEAM1 was several days shorter than that of MED [5]. This provided MEAM1 a numerical advantage that helped it excluded MED over the course of several generations. Second, several studies have documented asymmetric reproductive interference between MEAM1 and MED [31, 35]. Although MEAM1-MED crosses produce virtually no viable offspring [36], MEAM1 males are more aggressive than MED males in courting females of both species; as a result, MEAM1 males interfere more with intra-specific mating attempts by MED than vice versa [35]. While the behavior of MED females is unaffected by the presence of MEAM1 males, MEAM1 females mate more quickly with their own species when MED males are present. Because *B*. *tabaci* is haplodiploid, fertilized eggs become female and unfertilized eggs become male; the inability of MED females to compensate for reproductive interference by MEAM1 males yields a male-skewed sex ratio that decreases MED population growth [31, 35]. Laboratory-parameterized simulations of MEAM1-MED competition reveal that while MEAM1’s growth and reproductive advantages are both important, the asymmetric impact of MEAM1’s reproductive interference on MED can itself produce competitive exclusion.

There are several ways in which our results and the findings described in the previous paragraph can be reconciled. Specifically, the competitive exclusion of MEAM1 by MED on cotton in our experiment could result from (1) the ‘performance’ (i.e., reproduction and/or development time) of MED on cotton being improved by long-term rearing on poinsettia; and/or (2) the performance of MEAM1 (in general, or on cotton specifically) being degraded by long-term rearing on cabbage. While we cannot definitively rule out any of these mechanisms, there are several reasons why the latter ‘general degradation’ explanation appears unlikely. Cabbage is a preferred host for MEAM1 [34], which feeds better than MED on cabbage [15]; when both were reared on cabbage, MEAM1 had a higher egg hatching rate, shorter development time, and higher survival rate [37]. Consistent with this, our work and other studies (fig 1C) [22, 23] find MEAM1 is competitively dominant on cabbage. This occurs irrespective of whether both species are reared beforehand on cotton [23], tomato [22], or different host plants (this study). If long-term rearing on cabbage had a generally negative effect on MEAM1, we would expect to see less-rapid competitive exclusion of MED; instead, our work found cabbage-derived MEAM1 competitively excluded MED in 75–100 days. By comparison, the five studies listed in Fig 1b found competitive exclusion of MED on cotton in ~155 days.

The second possibility is while that long-term rearing of MEAM1 on cabbage did not affect (and may well have improved) its performance on this plant, it did decrease its performance on cotton, and perhaps other, host plants. This scenario seems more likely than the previous one: similar negative cross-host correlations in performance have been observed in aphids [38] and a range of other insect species [2]. Whiteflies reared long-term on cabbage may, for example, improve their ability to circumvent *Brassica* defenses at the cost of reduced performance on non-*Brassica* hosts. The possibility of negative cross-host performance correlations in *Bemisia* was addressed by Liu et al. [39], who isolated cabbage-feeding MEAM1 on three host plants (*B*. *oleracea*, *C*. *sativus*, and *L*. *esculentum*) for 80 generations and then examined each subpopulation’s feeding performance on all three hosts. They found that the performance of the *oleracea*-specific MEAM1 subpopulation equaled or exceeded that of the *sativus*-specific and *esculentum*-specific subpopulations on all three host plants; in addition, neither the *sativus*-specific or *esculentum*-specific subpopulations had the best feeding performance on their natal hosts [39]. Although this work did not find negative cross-host performance correlations, it only addressed feeding and would not have detected tradeoffs manifested in growth, development time, or survival.

In addition to the possibility that long-term rearing on cabbage reduced the tendency of MEAM1 towards polyphagy, long-term rearing on poinsettia might have provided MED several adaptive or epigenetic advantages over other host plants. One potential advantage might involve increased tolerance of phenolic-based plant defenses [40]. Whitefly fitness negatively correlates with phenolic levels in both tomato [41] and cotton [42], and phenols are the only secondary compound found in poinsettia phloem [43]. Although poinsettia and cotton both invest in phenolic defenses, a comparative analysis found that total phenols were 6x greater in poinsettia than cotton [17]. Long-term rearing on a high-phenol host plant like poinsettia may select for or produce epigenetic changes resulting in [44] whiteflies tolerant of phenolic concentrations far higher than those typically found in cotton, helping to improve their performance on this host plant.

Long-term rearing on poinsettia may also select for whiteflies with high rates of phloem consumption. Poinsettia is a relatively low-quality host plant, with foliar C:N ratios substantially higher than those of cotton [17]. *Bemisia* population growth is positively correlated with plant nutritional quality even though phloem consumption rates are higher on low-nitrogen plants across a range of host plant species [40]. If poinsettia does select for individuals with that rapidly feed on and process phloem, this adaptation may prove beneficial on a range of host plants. Given the role played by asymmetric reproductive interference in the MEAM1-MED interaction on cotton [31, 35], it is also possible that poinsettia-derived MED differ in some aspect of their mating behavior. Long-term rearing on poinsettia might select for MED males that are particularly aggressive in their courting behavior, for instance, or might favor MED females with a stronger preference for males of their own species. These latter possibilities are intriguing; there is no evidence for them, however, and no apparent rationale for why such changes would occur specifically on poinsettia.

Regardless of which species (MEAM1 or MED) was responsible for our results, or whether adaptation or epigenetic changes underlies them, we found that the outcome of interspecific herbivore competition can be altered by the natal host plants of one or both herbivore species. The host plant on which an interaction occurs is well-known to affect the outcome of competition, and it has recently been shown that the offspring of herbivores reared on different host plant varieties do better on ‘their’ variety [9]. By contrast, we find evidence for altered performance on a different host plant species that persists over several generations. This result, although novel, may be predictable: offspring are affected by parental food quality even when the two generations are reared on different host plants [45]. Although host plant adaptation is the most logical explanation for our results, it is worth noting that research in both plants [12, 13, 46] and animals [11] has found that maternal effects can persist into at least the third generation. Our work lends further support to research showing how an organism’s ‘neighborhood’ can affect its interactions with other species [7, 8], and suggests that these neighborhood effects may be wider-ranging, longer-lasting, and more consequential than previously anticipated.
